# Corticothalamic feedback locally modulates network state

**DOI:** 10.1038/s41598-025-05592-y

**Published:** 2025-07-01

**Authors:** S. Borbély, A. Zalatnai, É. Gulyás, V. Balogh, M. Csernai, P. Barthó

**Affiliations:** 1 Sleep Oscillations Research Group, HUN-REN Research Center for Natural Sciences, Budapest, Hungary; 2 HUN-REN Institute of Experimental Medicine, Budapest, Hungary

## Abstract

As the cortex receives most of its input from the thalamus, cortical layer 6 (L6) in turn sends a massive glutamatergic projection back to the corresponding thalamic areas. L6 feedback is morphologically and physiologically distinct from driver excitatory inputs, being, according to Sherman and Guillery, a modulatory pathway, akin to classical modulators. Here we tested this hypothesis by examining the effect of L6 corticothalamic activation on thalamocortical oscillations and network state, using extracellular recordings and optogenetic stimulation. We found that different patterns of L6 activity can promote the transition to both light- and deep sleep-like states, as well as to desynchronized activity. Low frequency L6 activation entrained sleep spindles in a temporal manner, while tonic L6 activity abolished spindling and promoted delta-rich sleep. Stronger L6 activation desynchronized the network, inducing gamma oscillations. These changes were confined to the activated thalamocortical circuit, suggesting that corticothalamic feedback acts as a local modulatory subsystem.

**Supplementary Information:**

The online version contains supplementary material available at 10.1038/s41598-025-05592-y.

## Introduction

The thalamus is the main source of information to the neocortex. Almost all sensory information passes through it during wakefulness or gets blocked during sleep. Yet, information flow through the thalamus is far from being unidirectional, as layer 6 of the cortex provides a massive feedback to the corresponding thalamic nuclei, that exceeds thalamocortical projections by an order of magnitude^1^. As high as 30–50% of synapses in the dorsal thalamus originate from L6 corticothalamic (L6CT) cells^2^, a subpopulation of pyramidal cells in L6^3^. The exact function of the corticothalamic feedback is still debated. The most likely role is the top-down fine tuning of sensory and other inputs at the thalamic level, for example during selective attention^4^.

According to Sherman and Guillery’s hypothesis, excitatory inputs in the thalamus fall into two categories: drivers, and modulators^5,6^. The former, originating from subcortical sources, or layer 5 of the cortex, reliably transmits information to thalamocortical (TC) cells, ensuring the classical relay function. The latter, the L6 corticothalamic projection, is more akin to classical modulatory systems, such as cholinergic or noradrenergic, and its role is the modification of the state of thalamic cells. In vitro experiments support this idea^7–9^, also numerous studies have shown the modulatory effect of L6CT neurons on sensory responses^10–17^. However, the criteria of a true modulatory subsystem involve the ability to influence the general network state, a question that has not been addressed yet.

The thalamocortical circuit takes a major part in the generation of sleep oscillations, especially delta and spindle rhythms^18,19^. L6CT cells, providing ionotropic excitation to both thalamic reticular and relay nuclei^7,20^, with a metabotropic component at the latter^21–23^, is well positioned to influence these oscillations. Corticothalamic feedback indeed provides spatial synchronization of sleep spindles^24,25^, and has been implicated in the termination of spindles^26^.

In this study, we explored how corticothalamic activation affects thalamocortical oscillations at different states of alertness using extracellular single, multiunit and local field recordings combined with optogenetic stimulation in the L6 corticothalamic-specific NTSR1-cre mouse line. The stimulation patterns were aimed to emulate naturally occurring slow wave, as well as desynchronized cortical activity. We found that different patterns of L6CT activity can promote transitions between activity patterns typically associated with light sleep, deep sleep, and desynchronized state. These state changes were specific to the activated thalamocortical circuit, suggesting that the corticothalamic feedback acts as a local modulatory subsystem.

## Results

**Fig. 1:**
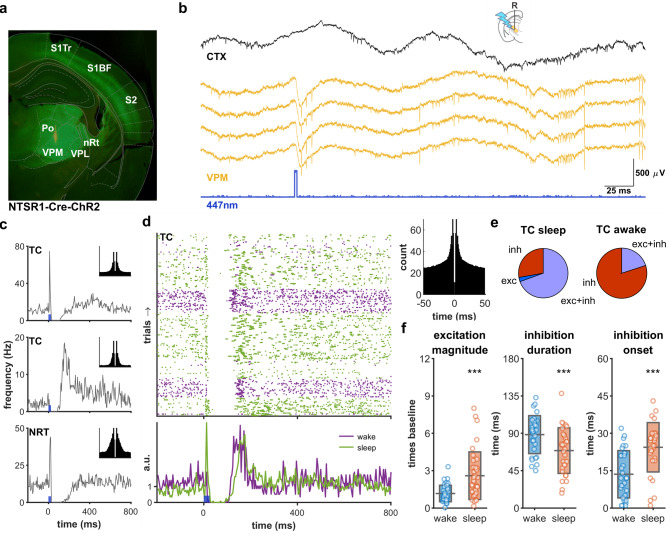
Effect of corticothalamic activation on thalamic cells is arousal dependent (**a**) Fluorescent micrograph from NTSR1-ChR mouse. Channelrhodopsin is expressed in somata (layer 6) and dendrites (layer 4-5a) of corticothalamic (CT) cells, as well as their axons in layer 4-5a, as well as in the thalamus. (**b**) Example ventral posterior thalamic response (tetrode recording) to a brief (2 ms, 4mW) light pulse to local CT axon terminals under urethane anesthesia. Cortex (S1) is not aligned topographically. (**c**) Typical responses (PSTHs) of thalamic units to brief (2 ms, 4mW) CT stimulation (insets: auto-correlograms). Thalamocortical (TC) cells either responded with brief excitatory peak followed by inhibition (top), or inhibition only (middle), and rebound, while thalamic reticular (nRT) units (bottom) usually showed excitation, followed inhibition. (**d**) Sleep/wake dependence of excitation/inhibition profile in a TC cell (rasterplot and normalized PSTH). Note the missing excitatory peak during wake state. (**e**) Ratio of units showing inhibition only is higher during wakefulness than during sleep. (**f**) In wake state, the excitatory peak is smaller (left), inhibition is longer (middle), and has a shorter onset (right) than during sleep. S1: primary somatosensory cortex; S2: secondary somatosensory cortex; VPM: ventral posteromedial nucleus of thalamus; VPL: ventral posterolateral nucleus of thalamus; Po: posterior nucleus, nRT: thalamic reticular nucleus.

For optogenetic manipulation of the corticothalamic feedback system we used the NTSR1-cre mouse line that selectively expresses Cre-recombinase in thalamically projecting layer 6 pyramidal cells^3^. Crossing these with the Ai32 reporter line (see methods) produced double transgenic NSTR1-ChR2 animals, that expressed channelrhodopsin-2 in both somata and axons of layer 6 corticothalamic (L6CT) cells (Fig. 1a). Local field and unit recordings were performed in the primary somatosensory thalamus (ventral posterior medial/lateral, VP), and primary somatosensory cortex (S1), using silicon probes for 9 urethane anesthetized mice and wire tetrodes or stereotrodes for 23 chronically implanted animals (Fig. 1b).

The behavioral state of the animals was classified into awake, slow wave sleep (SWS), and REM sleep. Though human sleep staging cannot be directly applied to rodents, slow wave sleep has been further subdivided to spindle-rich epochs, resembling human stage 2. sleep, and delta-rich epochs, resembling human stage 3. sleep. For the sake of conciseness, they will be referred to light and deep sleep, respectively (Fig S1).

### Sleep/wake dependent effect of corticothalamic activation on thalamic cells

Besides their direct projection on excitatory TC cells, L6 corticothalamic axons also send a collateral to the inhibitory cells of thalamic reticular nucleus (nRT). Our first aim was to determine how the excitatory and inhibitory components form the net effect of activation of corticothalamic feedback in the thalamus during sleep and wakefulness. Multiple single unit recordings were made from the primary somatosensory thalamic ventral posterior nucleus (VP). To ensure the proper topographic alignment of the stimulation, we targeted CT axon terminals in the vicinity of the recording site, instead of the corresponding cortical areas. Optic fibers were fixed to the chronically implanted wire tetrodes (n = 12 mice) or to parallel to the shanks of the silicon probe (32-channel, Neuronexus, Buzsaki32) used in the anesthetized mice (n = 9 mice). Brief (2-5 ms 447 nm, 1.5-4mW) pulses of blue light delivered every 5 s elicited visible response at the site of stimulation (Fig. 1b).

A total of 98 cells from freely moving animals were isolated following standard spike sorting procedures and cluster quality criteria (see Methods). While some units were classified as nRT axon terminals (n = 6) based on their narrow spike waveform and typical autocorrelogram (Barthó et al*.*, 2014), the large majority of units were classified as thalamocortical (TC) cells (n = 92). In response to a CT stimulation, TC cells typically showed a brief excitatory peak (8.7 ± 4.5 ms), followed by a period of inhibition (71 ± 26.3 ms, Fig. 1c, top), or only an inhibitory response, the latter two often followed by a rebound excitation (Fig. 1c middle). Thalamic reticular (nRT) units (n = 6) almost invariably responded with excitation followed by a silent period (Fig. 1c bottom).

The excitation–inhibition profile was dependent on arousal state. Of the chronically recorded TC cells showing significant responses, 38 cells produced enough spikes for comparison between both sleep and wakefulness. During sleep, in response to CT stimulation, majority of TC cells responded with a brief excitatory peak, an inhibitory trough, or both (2.7% only excitation; 69.6% excitation followed by inhibition;; 27.7% only inhibition), In awake state most cells responded with pure inhibition (20% excitation followed by inhibition; 80% only inhibition, Fig. 1d,e). The excitatory peak on the peristimulus-time histogram (PSTH) was larger during sleep (sleep: 2.7 ± 2, wake: 1.2 ± 0.7 vs ratio to baseline, *p* < 0.001). During sleep the inhibitory component was shorter (sleep: 70.3 ± 27.4 ms , wake: 86.3 ± 27.7 ms vs, *p* < 0.001), and had a longer onset time (sleep: 23.5 ± 9.7 ms, wake: 12.4 ± 9.2 ms, *p* < 0.001, Fig. 1f). Postinhibitory rebound was more pronounced during sleep (91.6% of cells rebounded, 2.7 ± 1.9 ratio to baseline), than during wakefulness (51.4% of cells 1.5 ± 0.7 ratio to baseline). The low number (n = 6) of nRT units recorded in both states did not allow similar analysis in a conclusive manner, but the reduced excitatory peak during wakefulness was present in all cells.

An additional 118 cells (n = 95 TC, n = 23 nRT) were recorded under urethane anesthesia. As it is characteristic to urethane, the animals spontaneously fluctuated between desynchronized (wake or REM-like), and synchronized (slow wave sleep-like) anesthesia patterns^27^. During these epochs, TC cells showed similar excitation/inhibition responses as during sleep and wakefulness, as summarized in Suppl. Table 1.

In summary, corticothalamic feedback exerts a sequence of excitation and inhibition on the thalamus. The excitation—inhibition profile is dependent on the sleep/wake state, as inhibition dominates during wakefulness, while in sleep there is a stronger excitatory component.

### Low-frequency CT activation entrains spindles

**Fig. 2:**
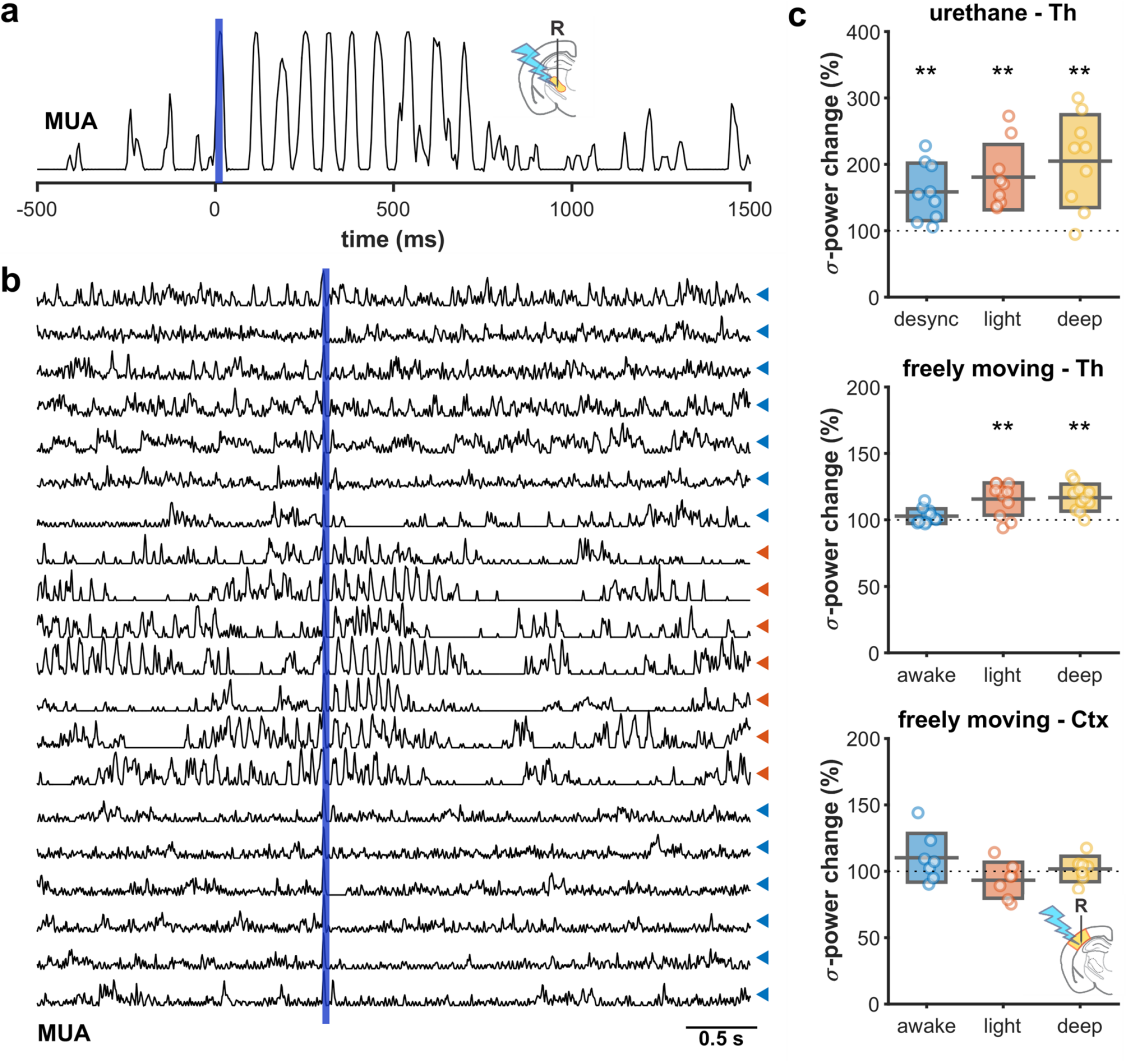
Spindle induction by corticothalamic activation (**a**) Example of a VP thalamic spindle evoked by a brief (5 ms) pulse to CT terminals under urethane anesthesia (smoothed thalamic multiunit activity). (**b**) Same stimulus across several trials. Spindle induction is most pronounced during spindling (light sleep-like) periods. blue triangles: desynchronized state, red triangles: light sleep-like state (**c**) Sigma power changes after CT stimulation (0.5–2 s) across brain states. Top: Thalamic stimulation and recording under urethane, spindle induction is most effective during synchronized anesthesia stages. Middle: Similar arrangement under unanesthetized conditions, stimulation of CT axon terminals in the thalamus evokes spindling during light and deep sleep. Bottom: somatic stimulation of CT cells is largely ineffective in inducing spindles (both stimulation and recording sites were in S1 cortex). Thalamic and cortical sites were not precisely aligned. Boxes denote mean and standard deviation. Significance denoted across all figures: *—*p* < 0.05, **—*p* < 0.01, ***- *p* < 0.001.

It is generally assumed that L6 corticothalamic feedback is the agent for the temporal coupling between synchronized cortical activity (up-down states, or K-complexes in humans) and sleep spindles. Therefore, we examined, if single pulse stimulations of L6CT could elicit spindles in either the cortex or thalamus. Under urethane anesthesia, brief stimulation of CT terminals reliably evoked spindles in both the VP thalamic (Fig. 2a) and S1 cortical multiunit activities. The likelihood of inducing spindles depended on the ongoing network state (Fig. 2b). characteristic to urethane, the animal spontaneously fluctuates between desynchronized, light (spindling) and deep (delta) sleep-like anesthesia patterns^27^. Spindles could be evoked with high probability during light- and deep sleep-like levels (80.8%, 104.9% sigma power increase, respectively), but less during desynchronized epochs (58.6%), (Fig. 2b,c). Under anesthetic-free conditions, the effect was less pronounced. By stimulating the L6CT axon terminals locally in the VP thalamus (Fig. 2c middle), sigma power showed a small, but significant increase in light- and deep sleep (15.7% and 16.8%, respectively), and no significant change during wakefulness. In another set of experiments, both the stimulation site and the evaluation of spindle power was in the primary somatosensory cortex (Fig. 2c bottom). Here we found no significant change in sigma power in either state. Various other stimulation parameters were tested (0.5–4 mW intensity, 2–160 ms duration) , none of them not producing overall significant effect.

**Fig. 3:**
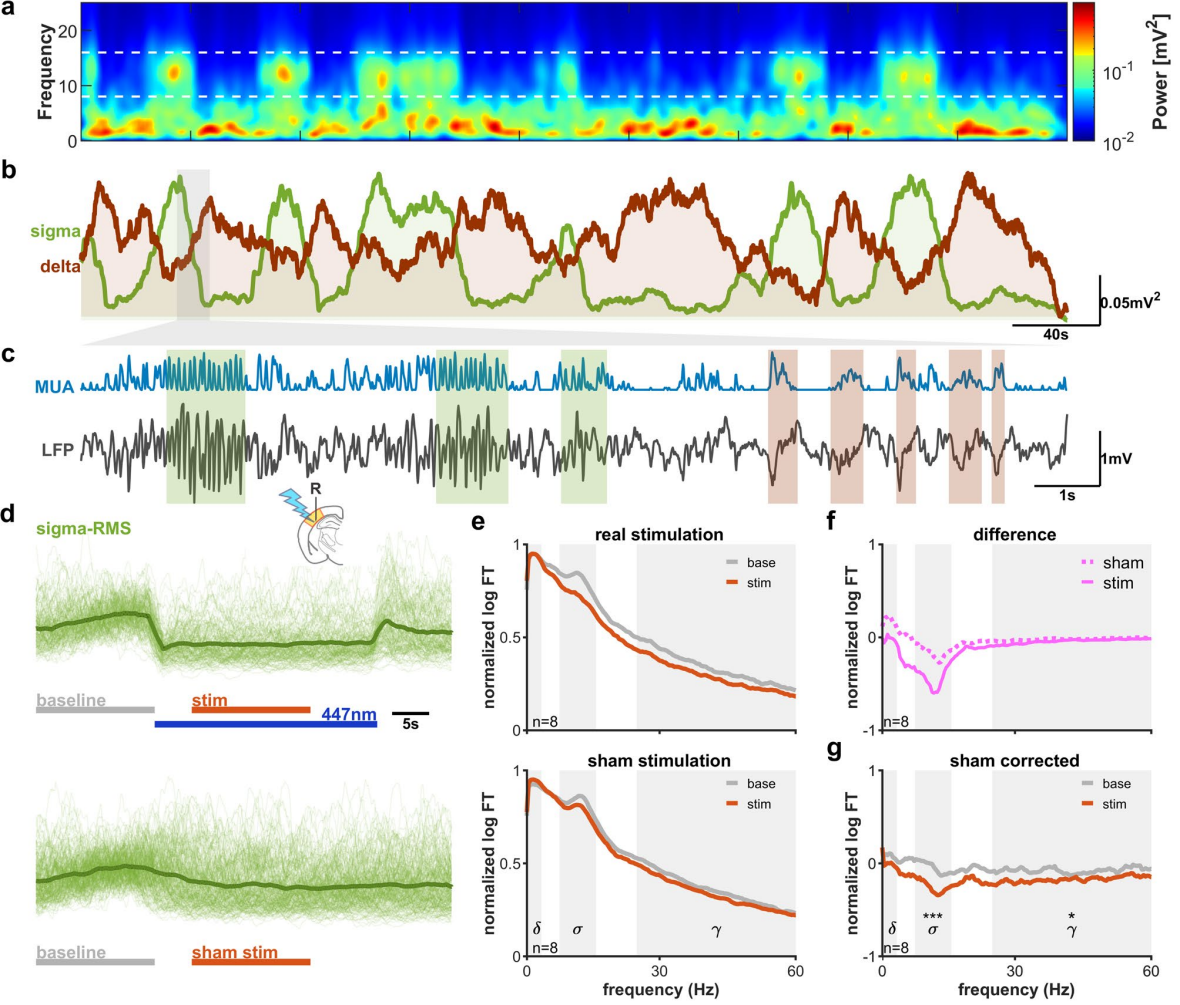
Sham correction for infra-slow oscillation (**a**,**b**) Spontaneous alternations of light and deep sleep epochs on the wavelet spectrum (**a**), and sigma and delta power (**b**) recurring every ~ 50 s. (**c**) Local field potential and smoothed multiunit activity during a transition (gray patch on panel (‘**b**’) from light to deep sleep. Green patches: spindles, orange patches: delta waves. (**d**) Analysis of prolonged stimulation data during infra-slow oscillation. The stimulus in this figure is for illustrative purposes only. Trials classified as light sleep in the baseline period were selected for the analysis. Baseline (gray line) period is compared to stimulated period (red line), omitting the stimulus onset. The stimulation causes a marked decrease in sigma power (**d**, **e** top). However, the spontaneous change from light to deep sleep also causes a decrease in sigma power (**d**, **e** bottom, sham stimulus). Traces are normalized to baseline. (**f**) Power difference between baseline for actual stimulation (solid line) and sham stimulation (dotted line). (**g**) In this example sigma power decrease in response to the stimulus is still significant after sham correction.

**Fig. 4:**
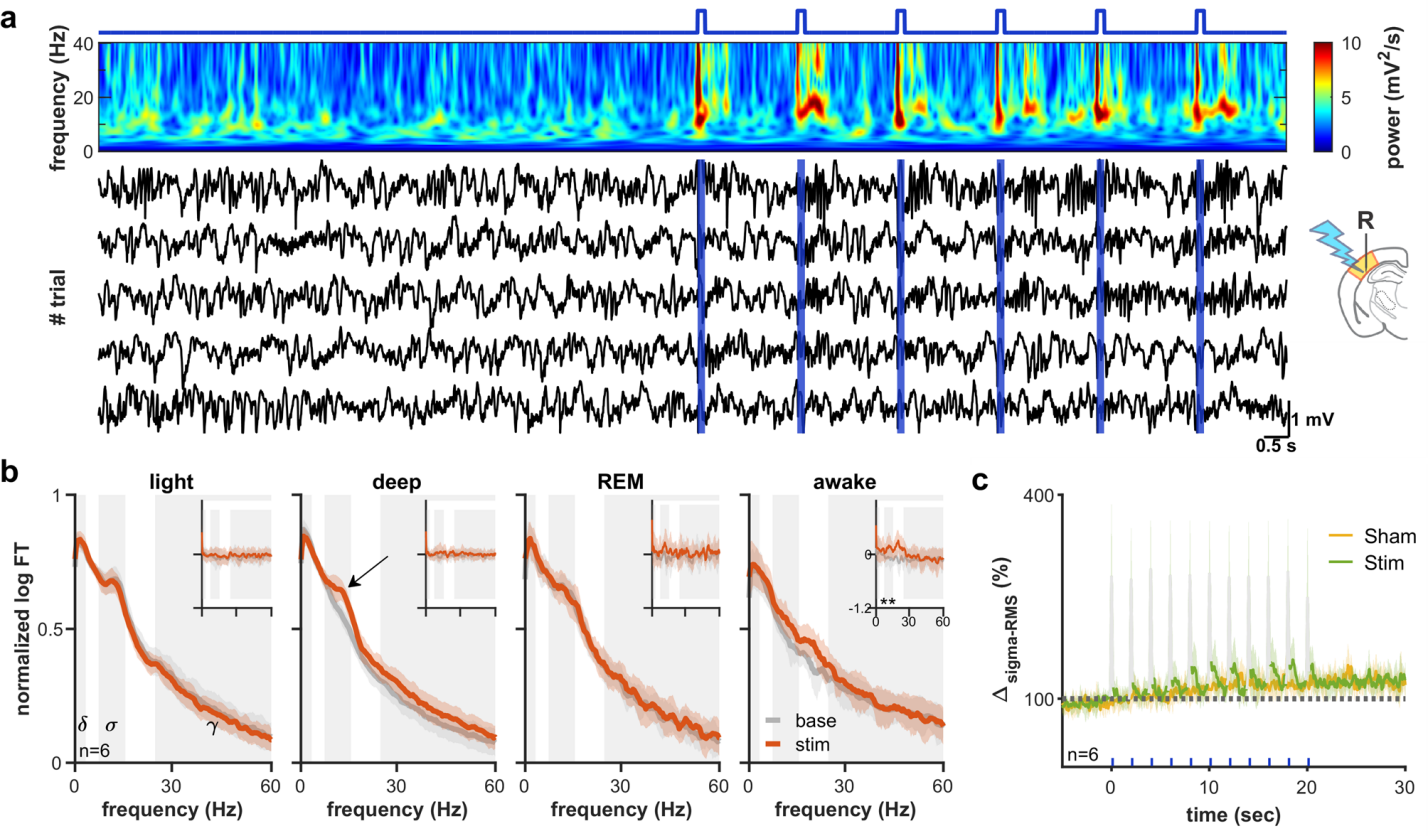
Low frequency CT stimulation temporally synchronizes spindles (**a**) Example 0.5 Hz (5 ms, 4 mW) stimulation trials of L6 corticothalamic cells during deep sleep. Increased spindling is apparent on both the wavelet spectrum (top) and the individual traces (bottom) of cortical LFP. The apparent paucity of delta power on the wavelet is due to the 1/f normalization. (**b**) Power spectra before (gray) and during (red) the stimulation during wake, light- deep-, and REM sleep epochs. Sham-corrected power spectra are shown in insets. Despite the increase in sigma power during deep sleep on the non-corrected spectrum (arrow), the sham corrected spectra reveal a lack of significant effect. (**c**) Relative sigma power during sham and real stimulation during deep sleep trials. Sigma power transiently increases after each CT stimulus but does not significantly differ from sham over the whole stimulus duration. Parts of the trace affected by stimulus artefacts are greyed out.

**Fig. 5:**
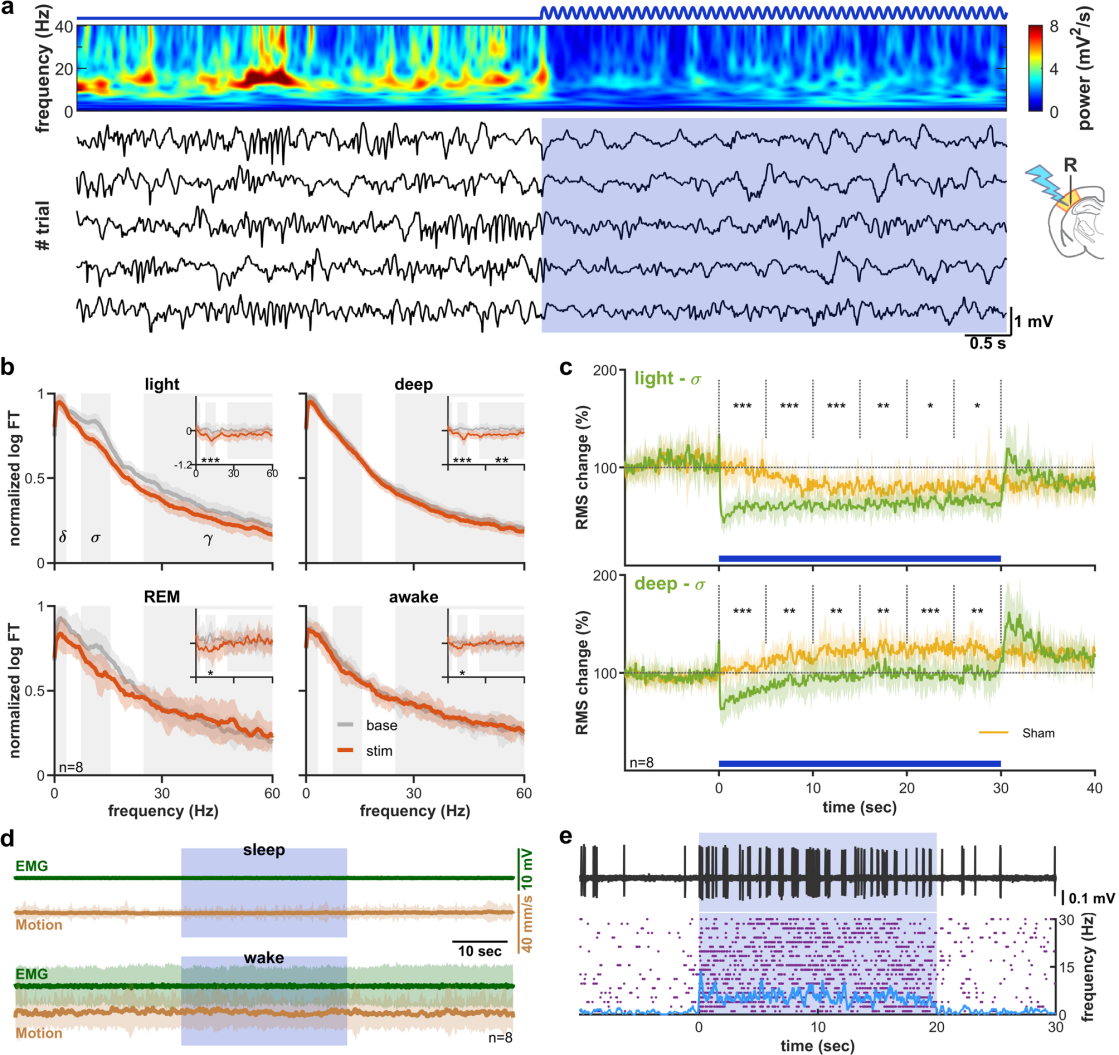
Tonic corticothalamic activation induces deep sleep (**a**) Example trials and mean wavelet of cortical LFP with prolonged weak tonic stimulation (300 Hz, 4 mW) during light sleep. Note the disappearance of sleep spindles during the stimulus. (**b**) Power spectra before and during stimulation for all four sleep stages (pooled data of 8 animals). (**c**) Relative sigma power during stimulation compared to sham stimuli in light (top) and deep sleep (bottom). (**d**) Mean EMG and camera-recorded motion show no apparent behavioral change during the stimulus. (**e**) Example trace (top), rastergram and PSTH (bottom) of a juxtacellularly recorded L6CT cell to a similar stimulus. Wavelet spectrum on (**a**) is 1/f normalized.

**Fig. 6:**
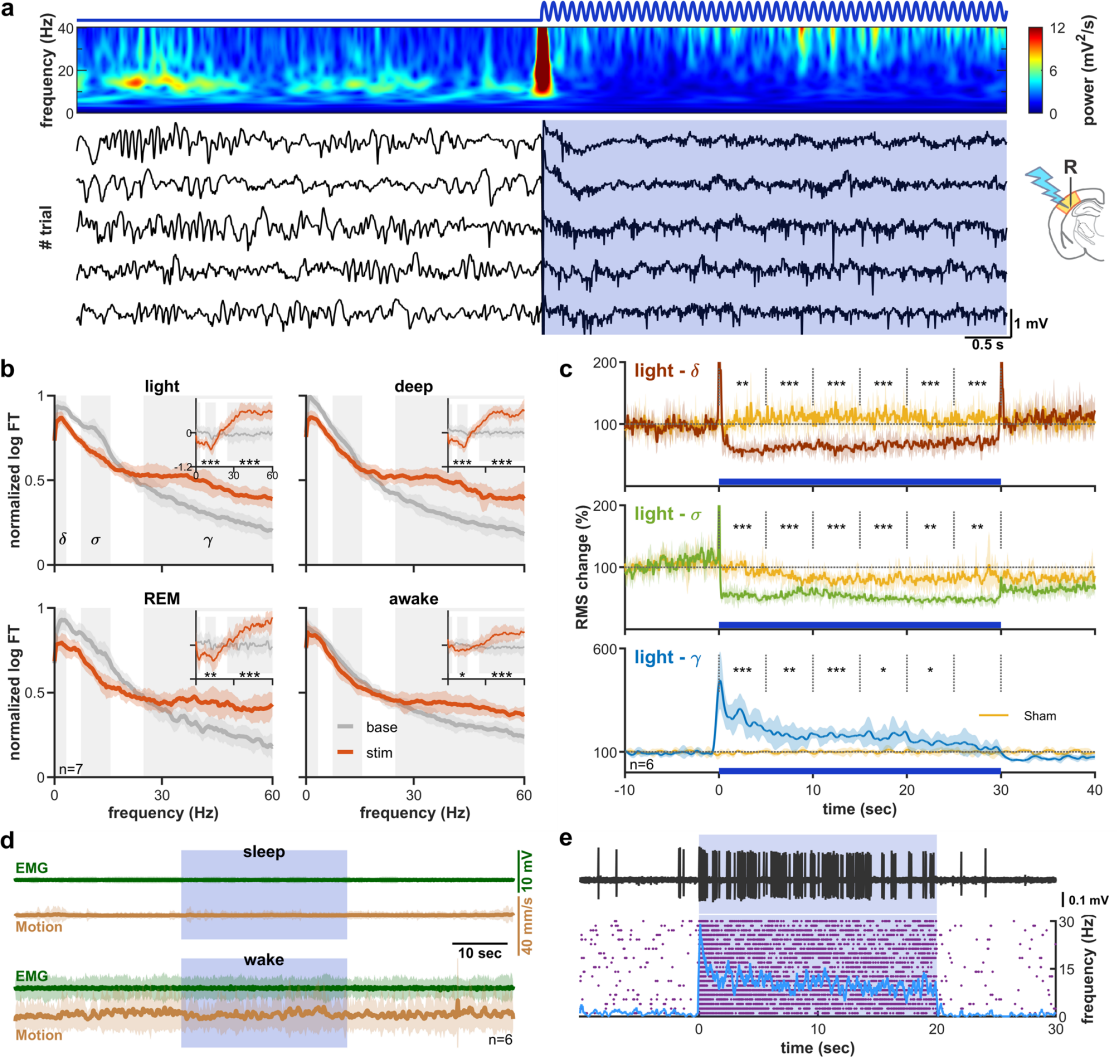
High intensity corticothalamic stimulation induces desynchronized state. (**a**) Representative cortical LFP traces before and during stimulation (300 Hz, 5.26 mW). Note the disappearance of both delta waves and spindles, as well as the enhanced gamma activity during the stimulus. (**b**) The effect is significant across all four states of alertness (pooled data of 7 animals). (**c**) Delta, sigma, and gamma RMS changes throughout the time course of the stimulus. (**d**) Mean EMG and camera-recorded motion show no apparent behavioral change during the stimulus. (**e**) Response of a juxtacellularly recorded L6CT cell to the stimulus used in this protocol. Wavelet spectrum on (**a**) is 1/f normalized.

Since cortical up-states repeatedly occur at the slow frequency range during sleep, we tested whether prolonged, repeated pulse-like L6CT activation has a stronger effect on sleep spindles, contrasting to isolated pulses. 20 s long periods of stimulation were compared with a preceding equivalent baseline period. Trials were grouped based on baseline period’s being classified into awake, light, deep, and REM sleep groups. This, however, complicates the analysis; rodent sleep exhibits the so-called infra-slow oscillation (Fig. 3a–c), where spindle rich (light sleep) and delta rich (deep sleep) periods alternate with a frequency of ~ 0.02 Hz^28,29^. During prolonged stimulation protocols, the network state has a high probability of transitioning to a different state between the baseline and stimulation periods. Selecting trials with a baseline period in the light sleep state (base period on Fig. 3d) shows an apparent decrease in spindles during the stimulated period, even though this is merely due to the effect of the spindle rich epoch transforming to delta-rich epochs (Fig. 3d). Correcting the power spectra for those of sham trials reveals a decreased, but still significant effect in this case (Fig. 3e–g). Therefore, all analysis for Figs. 4, 5, 6 will include sham-corrected power spectral analysis alongside the raw spectra.

To ensure exact topographic alignment between the thalamic and cortical components of the circuit, we assessed the effect using the local field and multiunit activity at the stimulated cortical site, instead of the connected thalamic areas. Since spindles, and to a large extent up- and down states are concomitant across connected cortical and thalamic areas, cortical rhythms reflect the activity of the whole thalamocortical loop. Periodic corticothalamic stimulation at 0.5 Hz (2 ms pulses, 6.8 mW intensity) produced an apparent locking of sleep spindles to the stimulus (Fig. 4a). Examining the effect across different stages of sleep and wakefulness showed that this stimulation significantly increased sigma power during deep sleep (Fig. 4b). Correcting for the sham stimuli, however, revealed that the effect was mainly due to the infra-slow oscillation (Fig. 4b insets, Fig. 4c). On the other hand, though the overall sigma power did not increase compared to sham stimuli, spindles were temporally phase locked to repeated L6CT activation. Therefore, although brief, single activation of the L6CT system appears to have little capability to induce spindles, repeated pulses, mimicking cortical activity during slow oscillation, have a strong effect on temporal synchronization of spindles.

### Tonic corticothalamic stimulation reduces spindling

Next, we tested how prolonged tonic activation of L6CT cells affects thalamocortical oscillations. To elicit moderate tonic firing without inducing a depolarization block in the target cells, we used a 30 s long, 300 Hz sinusoidal stimulus at 3.98 mW in S1 cortex (Fig. 5a). This intensity was able to elicit sustained tonic firing of L6CT cells at 13.58 ± 3.82 Hz (0.58 ± 0.09 Hz baseline), as verified by juxtacellular recordings in a separate set of experiments (Fig. 5e). Similarly to the previous section, for the sake of exact topographicity, both stimulation and recording sites were in primary somatosensory cortex.

Tonic CT activation during light sleep abolished spindling (Fig. 5a,b), giving way to delta oscillation throughout the length of the 30 s long stimulus. Stimulation during deep sleep caused no apparent power spectral change (Fig. 5b), however, after sham correction for the infra-slow oscillation, we found a significant decrease in sigma band in both light and deep sleep (Fig. 5b, insets). This is explained by the power changes during the stimulus (Fig. 5c). Due to the infra-slow oscillation, trials selected for light sleep in the baseline period tend to transition to deep sleep during the stimulation period, with a decrease in sigma power (Fig. 5c upper yellow trace). Nevertheless, tonic CT stimulation reduces spindling more than expected by the spontaneous state change (Fig. 5c upper green trace). Trials during deep sleep, on the other hand, tend to spontaneously transition to light sleep during the stimulation period, which is prevented by CT stimulation (Fig. 5b right inset, Fig. 5c lower panel). Sigma power reduction was significant both during light and deep sleep (*p* < 0.001), and to a certain degree, during wakefulness and REM sleep (*p* < 0.05). During slow wave sleep states there was a small, but significant decrease in gamma power (*p* < 0.01), and a reduction of delta during REM sleep (*p* < 0.05). To exclude possible volume conducted components of the LFP, we repeated the analysis on the smoothed cortical multiunit activity (Fig S3a), yielding similar results.

Such robust changes in the thalamocortical rhythms may reflect changes in the underlying state of alertness. This was assessed by the frame-to-frame movement on the video recording, as well as the electromyogram. We found no significant change in the movement or the EMG during tonic stimulation as compared to baseline, either during sleep or wakefulness (Fig. 5d). Analysis of movements by brain state (deep-, light-, and REM sleep) did not result in a significant change either. In conclusion, tonic corticothalamic activation causes a shift from light to deep sleep, but does not change the sleep/wake state of the animal.

### High intensity tonic corticothalamic stimulation induces desynchronized state

Tonic stimulation at higher intensity, however, produced a strikingly different effect. Similar 30-s, 300 Hz sinusoidal light pulse at 5.26 mW intensity was delivered to primary somatosensory cortex. This stimulus intensity elicited sustained tonic firing of juxtacellularly recorded L6CT cells at 18.74 ± 5.13 Hz (difference from low-intensity tonic stimulation *p* < 0.01), in a separate set of experiments (Fig. 6e).

This stimulus caused an immediate desynchronization of the thalamocortical network, accompanied by the disappearance of spindles and delta waves, that lasted throughout the whole stimulus (Fig. 6a). There was a marked decrease in both delta and sigma and an increase in gamma power, which was significant across arousal states (*p* < 0.001). All changes were also significant after sham correction. The sigma and delta decrease persisted throughout the whole stimulus duration, while the increase in gamma power waned after ~ 20 s (Fig. 6c). Surprisingly, examination of the video recording and the EMG changes during stimulation revealed no significant change in the movement compared to baseline, either during sleep or wakefulness (Fig. 6d), nor did separate analysis of deep-, light-, or REM sleep. Analysis of the smoothed cortical multiunit activity (Fig S3b), revealed similar power changes to the local field potential. All in all, though high-intensity tonic corticothalamic activation completely desynchronizes the thalamocortical circuit, without causing an observable change in the sleep/wake state of the whole animal.

### Corticothalamic modulation is local

**Fig. 7:**
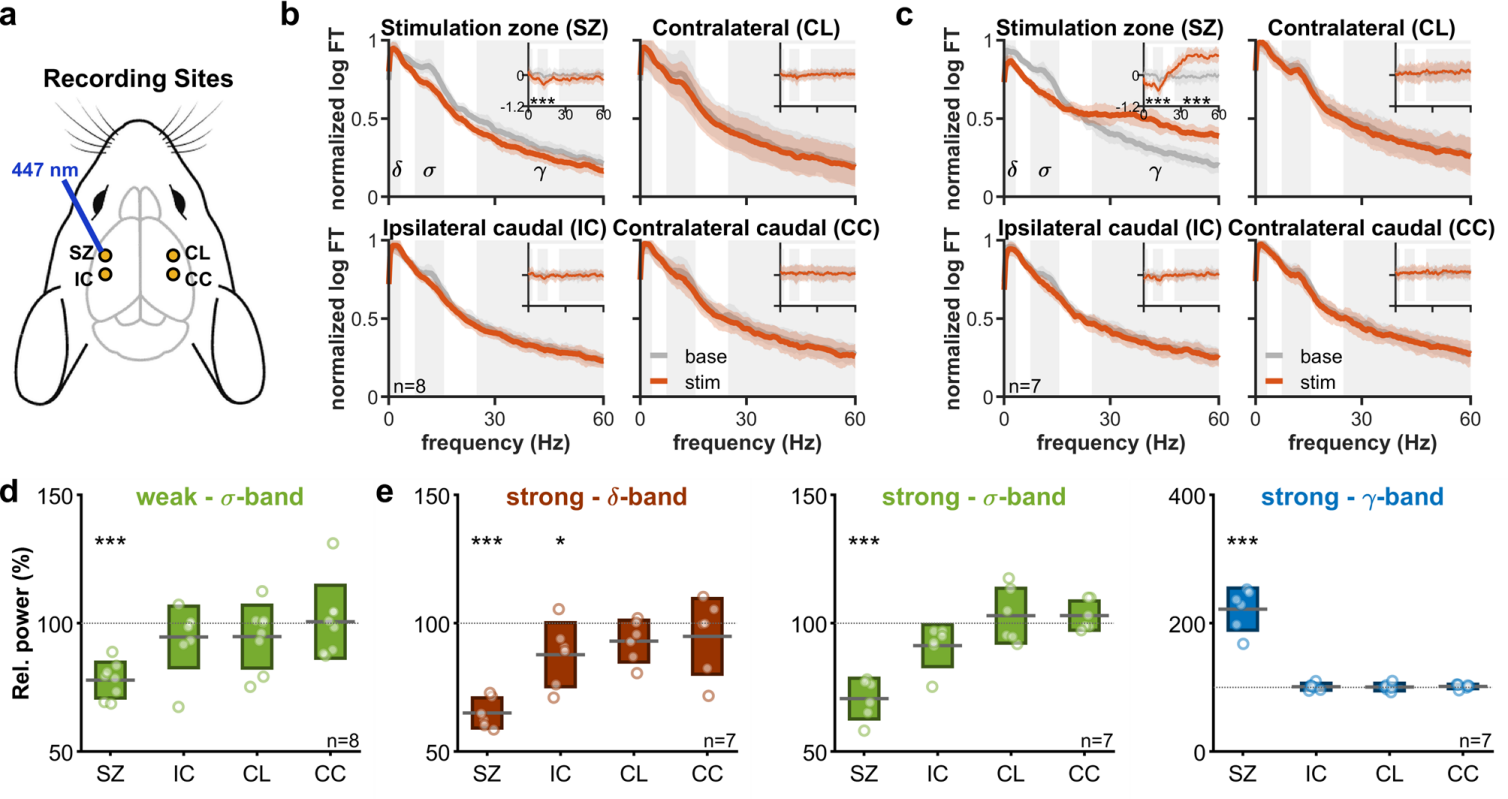
Corticothalamic modulation is local (**a**) Overview of the cortical recording sites. Besides the stimulated site in S1 (SZ), activity was recorded from S1 one mm caudally (IC), contralateral S1 (CL), and both (CC). (**b**) Effect of tonic stimulation at the stimulation site versus distal sites. The latter shows no significant effect on the sham-corrected spectrum. (**c**) Same as (**b**), with high intensity stimulation. Again, distal sites show no significant effect. (**d**) Relative sigma power change at different sites during tonic stimulation (**e**) Relative delta, sigma, and gamma power change at different sites during high-intensity stimulation.

To shed light on why the activation of L6CT cells induced profound changes in the local network activity but did not result in a change of sleep–wake state, we analyzed the activities recorded distantly (~ 1 mm posterior, contralaterally, or both) from the stimulation site (Fig. 7a). Considering the absorption of blue light in brain tissue, and the cone of influence, the illumination at this distance is negligible (see methods). Spectral analysis shows that during weak tonic CT activation the neighboring sites show only marginal changes in delta, sigma, or gamma powers on the uncorrected spectrum (Fig. 7b,d) and no change on the sham-corrected spectrum. Similarly, no effect was seen on the distant sites during the high-intensity tonic stimulation, either with or without correcting for the infra-slow oscillation (Fig. 7c,e). Analysis of the multiunit activity gave similar results (Fig S3c, d). These results suggest that corticothalamic modulation acts in a fashion confined to the affected thalamocortical circuit.

## Discussion

The aim of our experiments was to elucidate, how different patterns of L6 corticothalamic activation influence thalamocortical oscillations in various networks states, and whether they can alter the network state itself. At the single cells level, as expected, we found that corticothalamic activation exerts a mixed excitatory-inhibitory effect on thalamocortical cells. This profile was dependent on sleep/wake state, inhibition dominating during wakefulness, while during sleep the excitatory component was also pronounced.

The effect of activation of corticothalamic feedback at the network level was more complex. While brief pulses of L6CT activation had only a weak spindle inducing effect under anesthetic-free conditions, repeated stimulation at the slow oscillation frequency effectively synchronized spindling on the temporal scale. Tonic L6CT activity, on the other hand, resulted in an abolition of sleep spindles, shifting the network state from light to deep sleep. Higher intensity L6CT stimulation effectively desynchronized the thalamocortical circuit, causing a strong decrease in delta- and sigma, and an increase in gamma power.

Surprisingly, all these effects were to a high extent localized to the stimulated thalamocortical circuit. Even the strongest cortical desynchronization did not extend to cortical sites ~ 1 mm distant from the stimulation site, nor contralaterally. Even though the manipulations took place in the somatosensory system, none of the local state changes had an observable effect on the general level of arousal.

### Technical considerations

One possible pitfall of L6CT stimulation is the topography of the thalamocortical circuits. It is widely assumed that L6CT cells project roughly to the same area their column receives input from^30,31^, at least in the case of first-order nuclei. The finer details, e.g. if it is a closed or an open loop, and the extent of lateral inhibition via the CT collaterals to the nRT, are however not clear. Even with meticulously matching the regions, stimulating in the cortex and recording in the thalamus may result in dominant inhibitory, instead of excitatory effect, with only a minor difference in topographic alignment. Therefore, the analysis of network effects was based on cortical local field and multiunit activity at the stimulation site. Similarly, when analyzing thalamic responses, we included only the data where the L6CT axon terminals were locally stimulated, in the vicinity of the recorded neurons. 

We chose the sinusiodal stimulus for tonic activation after realizing that prolonged continuous illumination results in a depolarizing block in the stimulated cells. The 300 Hz sinusoidal stimulation prevented this, yet did not elicit phase-locking to the stimulus, as it is faster than the channerhodopsin inactivation time constant (~ 10 ms), resulting in a tonic activity confirmed by juxtacellular recordings. Question is, do the elicited L6CT firing rates fall within physiological boundaries? Though most studies use stimulation parameters comparable to our high-intensity tonic activation^10–12,14,16^, and often elicit gamma oscillations, we have to consider that L6CT cells are traditionally thought to be sparse firing^32,33^. A recent Ca^2+^ imaging study, however, reveal firing rates comparable to our juxtacellularly recorded cells^34^.

The third question is, whether axonal and somatic stimulation of CT cells produce comparable effects, especially regarding the CT axons’ collateral to the nRT. It has been shown that optogenetic activation of axon terminals produce backpropagating action potentials similarly to electrical stimulation^35^. We found the stereotypical excitation-inhibition sequence in TC cells with both cortical and thalamic stimulation, suggesting that the backpropagating action potential reliably invades the collateral to the nRT. In principle, the backpropagating route may cause a minimal delay in the firing of the CT terminals in nRT, but considering the axonal conduction velocities and distances we consider this negligible.

Another difference between somatic and axonal CT stimulation is the degree of convergence/divergence in the system, resulting in locally activating CT axons from a wider cortical area, than by cortical stimulation. In this respect, axonal stimulation has the advantage of entraining only those nRT cells that are connected to the collaterals of the stimulated CT fibers. Finally, due to the wide range of CT axonal conduction velocities^36–39^, axonal stimulation is likely to result in more synchronized postsynaptic potentials than cortical stimulation.

### Arousal dependent switch of CT effect

CT activation exerts a direct excitation on TC cells, followed by an indirect inhibition via the nRT, as described previously^40–43^. Our results showed a more variable excitation/inhibition profile on individual cells, mostly exhibiting the inhibitory, but only a minority showing the excitatory component. These differences may arise from topographic misalignment between CT and TC cells, but since our locus of stimulation was in the near vicinity of the recorded cells, this explanation is unlikely.

Our results were in accordance with studies where neuromodulators or brainstem stimulation altered the net CT effect^9,44^. One possible mechanism may arise from the timing differences in T-currents. TC cells express CaV3.1 channels, while in nRT CaV3.2, and CaV3.3 are found, with differing activation constants^45^. T-current found in nRT has an activation delay of tens of milliseconds compared to that in TC cells^46^. This difference provides a window of opportunity for excitation before the inhibitory component activates. During wakefulness both cell types fire tonically, and nRT units will respond fast to incoming CT excitation due to their compact membrane and stronger CT synapses^47^. This is in accordance with our results showing that during wakefulness, a smaller percentage of TC cells exhibits excitation to CT pulses than during sleep.

This state dependent switch of corticothalamic feedback, besides contributing to differently altering oscillations during sleep and wakefulness, may have a role in sensory processing as well. Thalamic burst firing, though most abundant during sleep, also occurs during inattentive wakefulness, as well as in sensory thalamic cells outside the attended area^48,49^.

### Is there a role of L6CT cells in spindle initiation?

Cortical activity has long been proposed to have a role in spindle generation and synchronization. Spindles often occur after K-complexes, indicating that either synchronous corticothalamic excitation or the concurrent indirect inhibition via the nRT during synchronized cortical activity can directly initiate the excitation-inhibition cycle required for spindling^50–54^. Electrical stimulation of the cortex can induce spindles^55^. Optogenetic stimulation of the nRT can also elicit immediate spindling^56,57^, probably due to rebound bursts after nRT inhibition^58,59^. Our experiments under urethane anesthesia seemingly confirmed this hypothesis, under natural sleep however, despite testing a wide range of stimulus intensities, we found only a weak spindle induction effect of corticothalamic stimulation.

This raises the possibility that the link between cortical up-states and sleep spindles is mediated by other corticofugal projections, such as the layer 5 drivers, or the projection-wise very similar layer 6b corticothalamic cells^60^. However, these project to higher order thalamic nuclei without giving off a collateral in nRT. Furthermore, higher-order thalamic nuclei are connected to different tiers of the nRT, where the cells do not fire rhythmic bursts at release from hyperpolarization (as opposed to their first-order connected counterparts)^61,62^. Nevertheless, layer 5 stimulation can induce a delta wave that may be followed by a sleep spindle^63^.

### L6 as a modulator

Our results show two kinds of state changes elicited by tonic corticothalamic activation; a transition from spindling to delta state, and complete desynchronization, accompanied by gamma oscillations. The question arising when interpreting these effects is, are they thalamic or cortical?

A possible mechanism of light to deep sleep transition in response to tonic CT stimulation is a prolonged hyperpolarization in TC neurons in response to tonic nRT activation^64^. Since the generation of spindles occurs only at a range of -60 to -65 mV^65^, any further hyperpolarization might switch the network to slow wave/delta mode. Indeed, tonic optogenetic stimulation of the nRT, reliably induces local deep sleep^66^, very similarly to our results with L6CT activation. A notable difference is, that the increase in slow and delta activity was more pronounced than in our experiments. They additionally reported a decrease of the arousal state, an effect our experiments did not elicit. This is most probably due to the different size of activated nRT area. Focal activation of S1 corticothalamic cells will influence a much smaller sector of nRT than direct nRT injection. Also, since they used vGAT-cre mice, higher order-, and intralaminarly projecting sectors were possibly affected in addition to the nRT tiers projecting to primary nuclei.

Thalamic inactivation by TTX or the selective T-channel blocker TTA-P2 also eliminated spindles, and decreased the frequency of slow waves^67^. Similar effects have been shown in^68^, where thalamic inactivation during wakefulness enhanced slow oscillation in the cortex. By tonic activation of the thalamus they found a pronounced cortical desynchronization, in line with our results, albeit with a less pronounced gamma component. The stronger gamma oscillations in our experiments may originate from the vertical projection of L6CT cells on fast-spiking interneurons^3^. Finally, since L6CT cells provide intracolumnar collaterals to other layers^69^, a purely cortical origin of the gamma component cannot be ruled out.

On the other hand, several studies point to the dominance of depolarizing effect of prolonged corticothalamic activation in the thalamus^42,43,70^. High-frequency stimulation of corticothalamic fibers activates metabotropic glutamate receptors, depolarizing thalamocortical cells, likely switching them from burst to tonic mode^43^. Similarly, glutamatergic inputs produce NMDA plateau potentials in distal dendrites (the preferred target of corticothalamic axons) of thalamic relay cells^70^. Also, repeated CT stimulation produces progressively facilitating ionotropic EPSPs, and slightly depressing IPSPs, in a frequency-dependent manner^42,71^.

Though cortical layer 5 was not directly stimulated, we cannot rule out their involvement in the observed effects. However, as similarly to the primary afferent drivers, their synapses in the thalamus lack metabotropic component, and show marked synaptic depression^72^, it seems unlikely that they could contribute to long-term depolarization of thalamic cells.

The complexity of underlying synaptic currents observed in vitro, aggravated by the recurrent organization of intact thalamocortical circuitry in vivo makes a clear cellular level explanation of our results difficult. We do not exactly know how the moderate-high frequency (6–50 Hz) stimulation protocols used in many of these studies translate to the prolonged tonic activation patterns used in our experiments. When attempting to use the common in vitro stimulation patterns in vivo, we often ran into circuit resonance effects resulting in paroxysmal activities, therefore they are not included in this paper. Nevertheless, a logical explanation is, that as low intensity tonic stimulation produces moderate firing in CT cells, the inhibitory component dominates resulting in reduction of spindles in favor of delta waves, whereas as CT firing rates get higher at higher stimulation intensities, the excitatory components become dominant, with concomitant desynchronization.

### Corticothalamic network state change is a local phenomenon

A general feature of modulatory systems is that a relatively small number of modulator neurons can induce a state change in widespread areas of the brain^73^. This is obviously not the case with the L6 corticothalamic feedback, where robust local network changes leave neighboring cortical areas and the arousal of the animal unaffected. This arrangement seems sensible, given the proposed role of L6CT feedback in selective attention, and also considering that sleep, though generally regarded as global, is often a local phenomenon^74,75^. Local changes in sleep and wakefulness may be the result of focused changes of classical neuromodulators, the homeostatic changes in local metabolites are also likely to play a role. For thalamocortical oscillations to occur, the thalamus and the cortex must act in synchrony (e.g. both cortical circuits and isolated TC cells are capable of oscillating at delta frequency), and L6CT feedback is ideally posed to set the thalamic state.

## Experimental procedures

### Surgery and recordings

#### Ethical considerations

Experiments were carried out in accordance with the Hungarian Act of Animal Care and Experimentation (1998, XXVIII) and with the directive 2010/63/EU of the European Parliament and of the Council of 22 September 2010 on the protection of animals used for scientific purposes. The study was designed and reported in accordance with the ARRIVE guidelines^76^. Experimental protocol was approved by the regional ethical committee (license number PEI/001/2290-11/2015 for our in vivo experiments). Efforts were made to minimize the number of animals used. Standard food-pellets and tap water were available ad libitum.

*Light–dark cycle* Mice were kept under a 12:12 h LD cycle (lights-on at 7:00 a.m.) in a temperature-controlled room at 22 ± 2 °C. Experiments were performed during the light phase (zeitgeber time 2–10) to ensure long periods of sleep recordings.

*Experimental animals* Our experiments were carried out on male NTSR1-Cre transgenic mice (GN200 strain, Gensat) crossed with Ai32 (Ai32.(RCL-ChR2(H134R)/EYFP)) reporter line resulting in NTSR1-ChR2 (n = 32) animals selectively expressing ChR2 by layer 6 corticothalamic cells. As control, NTSR1-cre (n = 4) mice were injected with a viral vector containing floxed YFP (AAV5.EF1a.DIO.eYFP.WPRE.hGH, UNC Vector Core), the effect of optogenetic stimulation carried out on these animals are shown on Fig S2. Animals were weighing between 18 and 30 g at the time of the surgery.

*Acute silicon-probe and juxtacellular experiments* For general surgical procedures see^56^. Briefly, the animals were anesthetized with urethane (1 g/kg; intraperitoneally), 1 × 1 mm craniotomies were made over the ventral posterior nucleus (VP) of thalamus (AP: -1 to -2.5; L: + 1.25 to + 2.5) and/or over primary somatosensory (S1) cortex (AP: 0 to -1; L: + 3 to + 4). During silicon-probe recordings a bilinear 32-channel or a 4-shank 32-channel NeuroNexus (Ann Arbor, USA) electrode was inserted and lowered into the brain (Br. AP -1.6, L + 1.8 at 3800 μm depth) at a speed below 0.2 mm/s. An optic fiber (125/250 μm; Thorlabs, Newton, New Jersey) was also inserted to the brain (Br. AP -1.6, L + 1.6 in 15–18° at 3600 μm depth) to illuminate the VP close to the electrode. After electrode insertion, we waited for at least 30 min before recording.

In the case of juxtacellular experiments linear 16-channel NeuroNexus silicon-probe was inserted and lowered into the somatosensory cortex at 1600 µm depth. Juxtacellular recording was performed by a glass micropipette (35–45 MΩ, 1 M NaCl and 2% neurobiotin; Biomarker Ltd, Hungary), positioned close (< 500 µm) to the linear silicon-probe. Micropipette was lowered into the brain by a Scientifica IVM Single in vivo micromanipulator (Scientifica, Uckfield, UK). To optogenetically activate L6CT cells an optic fiber was placed at the cortical surface and 0.2 Hz 20 ms ~ 10-15mW laser pulses were given continuosly to shortly activate tagged cells. L6 CT cells were collected in 900–1200 µm cortical depth.

*Chronic experiments* Electrode implantation was made under ketamine/xylazine anesthesia (ketamine: 100 mg/kg; xylazine: 4 mg/kg; intraperitoneally). During surgery, the depth of anesthesia was regularly assessed by the limb-withdrawal or corneal reflex, and additional dose of anesthetic was administered, if necessary. To record LFP and multiunit (MUA) activity, bundles of 25 and 50 μm tungsten stereotrodes (California Fine Wire; Grover Beach, USA), combined with an optic fiber were placed in the left and right anterior S1 (Br. AP 0.0, L ± 3.5, at 1000 μm depth), while 50 μm tungsten stereotrodes were placed in the left and right posterior S1 (Br. AP -1.0, L ± 3.5, at 1000 μm depth) as well as in the hippocampus (Br. AP -2, L -1.8, at 2200 μm depth). In experiments for chronic thalamic unit recordings, the same coordinates were used as in the acute ones (previous section). An EMG electrode was inserted in the neck musculature. Electrodes were connected to a Neuralynx EIB-16 electrode interface board (Neuralynx, Dublin, Ireland). During recovery period (10 days) animals were given analgesic medication in their drinking water (paracetamol) and monitored daily for signs of pain or inflammation. During each type of surgical procedures body temperature was continuously monitored and stabilized at 37 °C, using a heating pad. Craniotomy coordinates are based on the stereotaxic atlas of Paxinos and Watson (1986).

*Power density estimation* The mean of measured power intensity of optic fibers was 3.9 and 6mW for the weak and strong tonic stimulus patterns. The power density of 447 nm light at the layer 6 of neocortex was estimated as follows: first the size of illuminated area was calculated, using the numeric aperture (NA = 0.22) value of optic fiber and the refractive index (n_tissue_ = 1.36) of brain tissue^77^, according to the following formula:

$$\theta_{div} = \sin^{ - 1} = \frac{NA}{{n_{tissue} }} = 9.31^{ \circ }$$

where *Θ*_*div*_ is the half angle of divergence. The 100 µm optic fiber was inserted to the brain tissue at the depth of 300 µm, which means that the light should travel through 600 µm distance in order to reach the L6 CT cells. Using tangent trigonometric function, considering the *Θ*_*div*_ = *9.31°* half angle, the diameter of illuminated L6 tissue volume was 296.6 µm, with the area of 0.069 mm^2^. Second, the light power should be estimated at the level of L6 CT cells. According to previous data from literature^78^, the light power declines to ~ 5% of the initial value at the distance of 600 µm, resulting in 0.195mW and 0.3mW power for weak and strong tonic stimulation while the corresponding power density values are 2.83 and 4.35 mW/mm^2^, respectively.

Intensities for weak and strong tonic stimulation were selected during the pilot experiments to maximize effect purity. Post-hoc verification of the actual intensity delivered by the fiber revealed a small jitter (± 0.31 mW for weak, ± 0.41 mW for strong stimuli), which was way too small to cause overlap between the stimulus batteries.

### Data acquisition

All the LFP signal were recorded against a screw electrode implanted over the cerebellum which served as a reference. Silicon-probe and tungsten stereotrode recordings were made with Intan RHD2132 32-channel amplifiers, connected to an Intan RHD-2000 Evaluation Board (Intan Technologies, Los Angeles, CA). The output of the Roithner RLTMDL-447–100-5 laser device (Roithner Lasertechnik GmbH, Vienna, Austria) was controlled by an analog signal generated by an NI-USB6353 (National Instruments, Austin, Texas). All signals were sampled at 20 kHz and synchronized through the analog input of the Intan system. Juxtacellular signals were amplified and filtered (2000x, 0.1 Hz high-pass) with a MultiClamp 700B amplifier (Molecular Devices, San Jose, CA) and digitized by the Intan recording system. For optogenetic stimulation, blue (447 nm), 100–200 mW lasers were used (Roithner, Austria).

### Data analysis

*Preprocessing and clustering* Raw recordings were band-pass filtered between 0.1-7000 Hz. For multiple single unit analysis, spikes were detected and clustered by a semi-automatic clustering algorithm (‘‘KlustaKwik’’; available at http://github.com/klusta-team) followed by manual postprocessing. Auto- and cross-correlograms were inspected to verify the clustering procedure. The quality of spike clusters was estimated with the ‘‘isolation distance’’measure^79^. TC and nRT units were distinguished by spike width and autocorrelogram shape as described in Barthó et al*.*, 2014. In case of juxtacellular recordings, spikes were detected with a simple window discriminator. For multiunit analysis, spikes were detected from each electrode group, downsampled to 1 kHz and smoothed with an 11 ms moving average filter.

*Spectral analysis* Frequency analysis was performed on both the local field potential and the smoothed MUA. Wavelet transform between 0.1–40 Hz was done using the Morlet wavelet function^80^. Power spectrum was calculated as the log of the Fourier transform (Matlab *fft.m’* function) of each trial averaged for each animal, during baseline and stimulation, respectively. Sham corrected spectrum was defined as the ratio of power spectral change during real and sham stimulation. Delta- and sigma-RMS were calculated as an integrative of 0.3–4 Hz or 9-16 Hz band-pass filtered LFP, respectively.

*Sleep stage classification* Classification of recorded epochs according to sleep/wake state was done by a semi-automatic method based on machine learning followed by manual verification. The training of the classifier was based on a previously carefully analyzed dataset, verified by three experienced individuals. Namely, deep sleep was distinguished by its delta waves, high delta power in S1, as well as lack of sleep spindles and hippocampal theta activity. Light sleep was characterized by the prominence of sleep spindles (and a marked sigma power peak) in S1. REM sleep was discerned by the prominent hippocampal theta, with no concomitant muscle activity. Wakefulness was distinguished by the increased MEG baseline, elevated hippocampal theta, and decreased delta and sigma power. Some transitional epochs, showing no clear affiliation to either these states, were omitted from the analysis. The algorithm extracted delta-, sigma-, theta- and gamma-power of cortical/thalamic LFP, smoothed MUA, hippocampal LFP and EMG channels and classified them as awake, light-, deep-, and REM sleep respectively, in case of urethane anesthesia light, deep and desynchronized states, using a Tree classifier (Matlab Classification Learner App). The result of automated classification was manually verified. The animal was continuously monitored through a video camera during recording sessions. Frame- to frame movement was assessed by the difference between the center of weight of the animal.

*Statistics* Significant excitation on the PSTH was defined as the number of spikes during 0–20 ms after stimulation exceeding the 95% confidence interval of a Poisson distribution with mean based on an equivalent baseline period. Similarly, inhibition was considered significant when the number of spikes in the PSTH was below 0.05% during the 30–70 ms period post-stimulus. Spectral bands were compared with a paired t-test (baseline vs. stimulated). Homogeneity of variances and normal distribution of data were tested before statistical analysis. All data are presented as Mean ± SD (standard deviation of the mean) unless otherwise indicated.

### Histology

Following the recordings, animals were euthanized under deep ketamine/xylazine (ketamine: 200 mg/kg; xylazine: 8 mg/kg; intraperitoneally) anesthesia by transcardial perfusion with 0.9% saline, followed by fixative containing 4% paraformaldehyde and 0.1 M phosphate buffer (PB). After perfusion brains were sliced to 50 µm thick coronal sections with a Leica VT1200S vibratome (Leica Biosystems, Wetzlar, Germany). After further PB washes, sections were mounted in PB coverslipped with Vectashield (Vector Laboratories, Burlingame, California) and imaged using an epifluorescent Leica DM2500 microscope (Leica Microsystems, Wetzlar, Germany).

## Electronic supplementary material

Below is the link to the electronic supplementary material.

Supplementary Material 1

## Data Availability

The datasets used and/or analysed during the current study available from the corresponding author on reasonable request.
