# Supplementary material for: Corticothalamic feedback locally modulates network state
Source: Sci Rep. 2025 Jul 1;15:20606. doi: 10.1038/s41598-025-05592-y (PMC12214504; doi:10.1038/s41598-025-05592-y)
Supplement: Supplementary file 1 — Supplementary Material 1 [file 41598_2025_5592_MOESM1_ESM.docx]

|  | sleep | awake | urethane synchronized | urethane desynchronized |
| --- | --- | --- | --- | --- |
| excitation only (% of cells) | 2.7 | 0 | 16.6 | 4.7 |
| mixed excitation / inhibition (% of cells) | 69.6 | 20 | 57.6 | 36.9 |
| inhibition only (% of cells) | 27.7 | 80 | 25.6 | 58.3 |
|  |  |  |  |  |
| excitation magnitude^1^  (ratio to baseline) | 2.7 ± 2 | 1.2 ± 0.7 | 5.7 ± 12.4 | 1.7 ± 1.7 |
| inhibition start (ms) | 23.5 ± 9.7 | 12.4 ± 9.2 | 25.4 ± 21.8 | 18.5 ± 14.9 |
| inhibition duration^2^ (ms) | 70.3 ± 27.4 | 86.3 ± 27.7 | 69.7 ± 52.8 | 81.5 ± 51.3 |
| rebound (% of cells) | 91.7 | 51.4 | 83.3 | 67.8 |
| rebound magnitude^1^  (ratio to baseline) | 2.7 ± 1.9 | 1.5 ± 0.7 | 3.6 ± 5.2 | 1.6 ± 1.5 |

**Supplementary Table S1. Excitation / inhibtion profiles of TC cells under sleep / wakefulness as well as urethane synchronized / desynchronized states.** Data is based on peristimulus-time histograms following corticothalamic stimulation. 1: excitation magnitude was calculated by the PSTH peak 0-20 ms poststimulus, while rebound magnitude from the mean of the PSTH between 150-200 ms poststimulus. 2: stimulus duration in urethane was 20 ms compared to 2-5 ms in chronic animals, therefore inhibition duration is not necessarily comparable

**
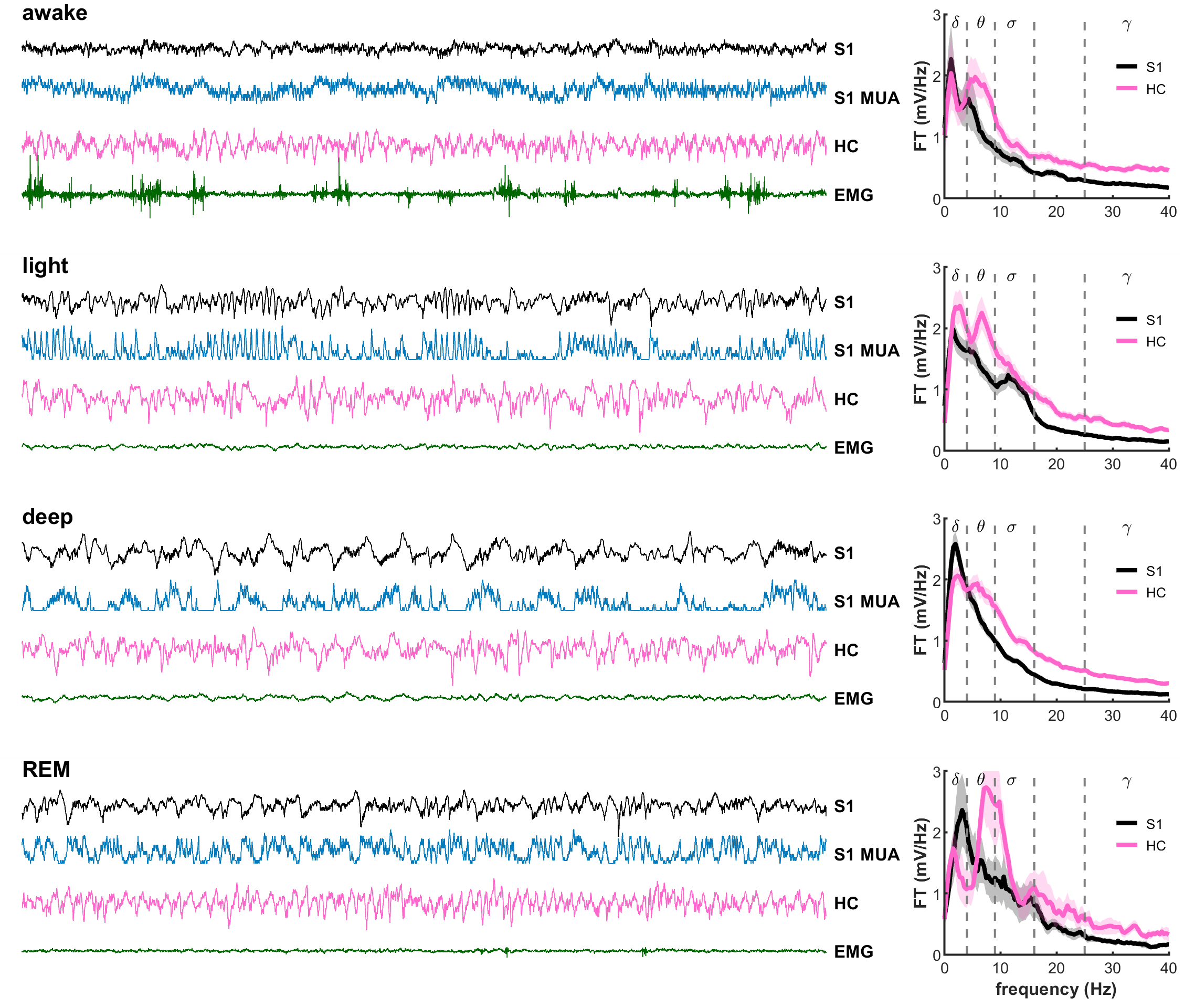
**

**Supplementary Figure S1**. **Overview of sleep scoring.** Classification was based on primary somatosensory cortex (S1) and hippocampal (HC) local field potential, S1 multiunit activity (MUA), and the electromyogram (left column). Spectral patterns characteristic for each state (right column) averaged from one recording session**.** Note the sigma peak in light sleep, and the hippocampal theta peak during REM.

**
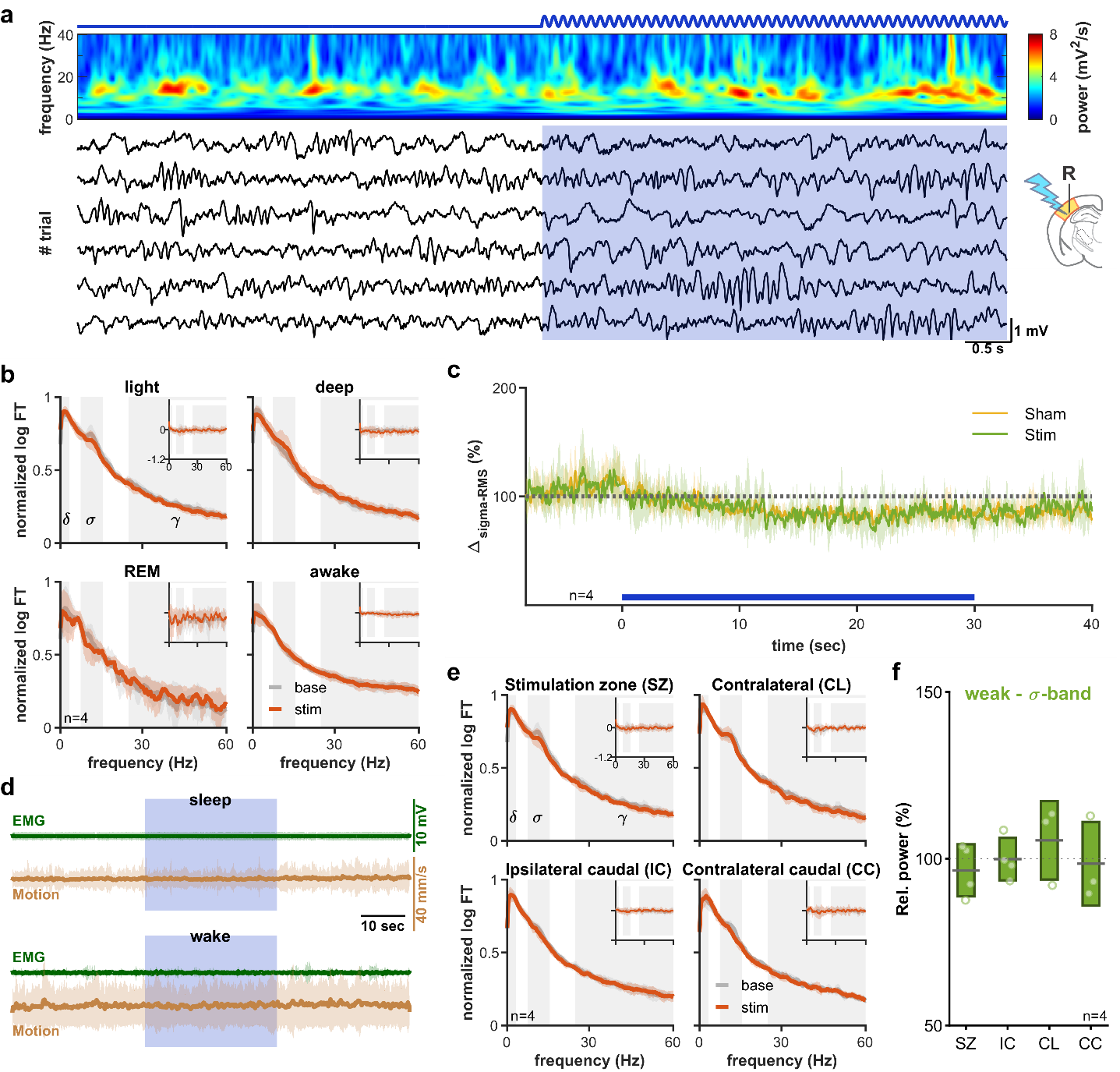
**

**Supplementary Figure S2. YFP control experiments** a-f) Same experimental arrangement to Fig 5 a-d, and Fig 7a on NTSR1-cre animals injected with floxed YFP vector. No significant effect was found on the sham corrected spectrum (b) or the RMS sigma power (c: example of light sleep trials). e, f) lack of effect on distal sites as on Fig 7.

**
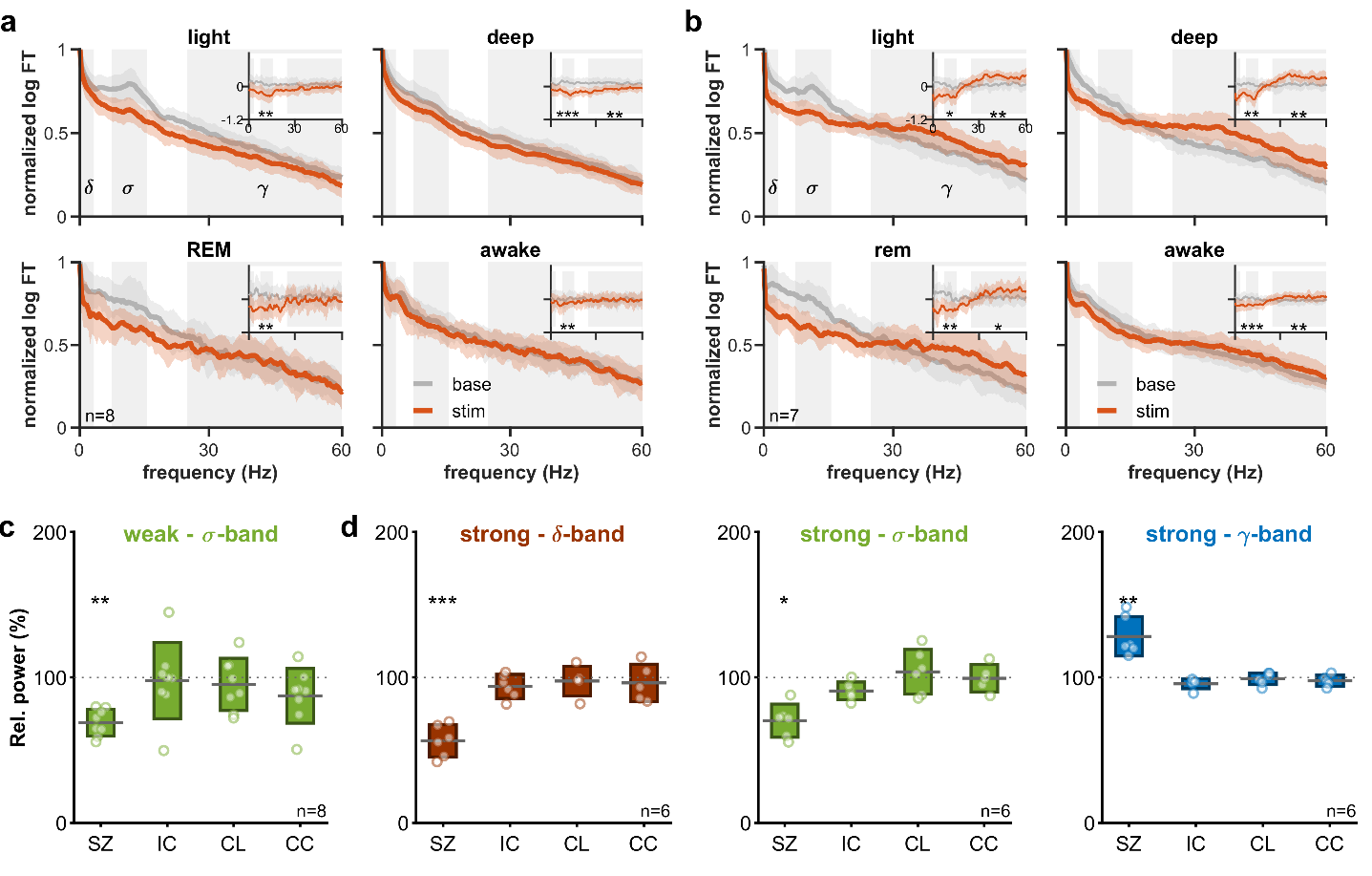
**

**Supplementary Figure S3. Analysis of multiunit activity is in line with LFP results.** a) Spectrograms of smoothed multiunit (sMUA) activity in response to tonic L6CT activation as in Fig 5. b) sMUA spectrograms for high-intensity tonic L6CT stimulation, as on Fig 6. d-e). Spatial extent of tonic (c) and high intensity tonic (d) L6CT activation as on Figure 7. (SZ: stimulation zone, IC: ipsilateral caudal S1, CL: contralateral S1, CC: contralateral caudal S1).
